# Gender differences in emotional response to the COVID‐19 outbreak in Spain

**DOI:** 10.1002/brb3.1934

**Published:** 2020-12-11

**Authors:** Lorena García‐Fernández, Verónica Romero‐Ferreiro, Sergio Padilla, Pedro David López‐Roldán, María Monzó‐García, Roberto Rodriguez‐Jimenez

**Affiliations:** ^1^ Clinical Medicine Department Universidad Miguel Hernández Alicante Spain; ^2^ Department of Psychiatry Hospital Universitario de San Juan Alicante Spain; ^3^ CIBERSAM (Biomedical Research Networking Centre in Mental Health) Spain; ^4^ Brain Mapping Unit Instituto Pluridisciplinar Universidad Complutense de Madrid (UCM) Madrid Spain; ^5^ Department of Psychiatry Instituto de Investigación Sanitaria Hospital Madrid Spain; ^6^ Infectious Diseases Unit Hospital Universitario de Elche Alicante Spain; ^7^ Facultad de Medicina Universidad Complutense de Madrid (UCM) Plaza Ramón y Cajal Madrid Spain

**Keywords:** acute stress, anxiety, COVID‐19, depression, gender differences

## Abstract

**Objective:**

We aim to explore the differential presence of symptoms of anxiety, depression, and acute stress between men and women during the COVID‐19 outbreak, and to study the relationship between these symptoms and two environmental variables, coexistence, and violence.

**Methods:**

We conducted a cross‐sectional study starting on March 29 to April 5, 2020, based on a national online survey using snowball sampling techniques. Symptoms of anxiety (Hamilton Anxiety Scale), depression (Beck Depression Inventory), and acute stress (Acute Stress Disorder Inventory) were assessed. Differences in the presence of symptoms and the relationship of coexistence and domestic violence were evaluated from a gender perspective.

**Results:**

Men showed significant lower mean (*SD*) in anxiety, depression, and acute stress levels than women [HARS, 14.1 (9.8) versus. 18.4 (10.2), *F* = 56.2, *p *< .001; BDI 3.4 (3.9) versus 4.5 (4.3), *F* = 16.6, *p *< .001, and ASDI 3.6 (2.9) versus 4.7 (3.1), *F* = 39.0, *p *< .001, respectively), as well as a weaker depressive syndrome (28.1% males versus 39.9% females, χ^2^ = 25.5, *p *< .001). In addition, an interaction Gender × Coexistence was found in anxiety (*F* = 56.2, *p *< .001) and acute stress (*F* = 3.52, *p *= .06) and, according to depressive symptoms, an interaction Gender × Violence was found marginally significant (*F* = 3.3, *p *= .07).

**Conclusions:**

Findings indicate that women present greater severity in symptoms of anxiety, depression, and acute stress. Moreover, loneliness and violence specifically worsen the emotional state in women. These results can undoubtedly guide better healthcare planning adopting a gender perspective.

## Graphical abstract

Our results indicate that women present greater severity in symptoms of anxiety, depression, and acute stress Moreover, loneliness and violence specifically worsen the emotional state in women.

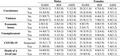

## 1 INTRODUCTION

The COVID‐19 outbreak appeared in December 2019 in China and rapidly spread worldwide, becoming an unprecedented traumatic event. This forced the World Health Organization to declare COVID‐19 outbreak a health emergency of international concern, and consequently, government entities in different countries, including Spain, took drastic measures to restrict the mobility of its inhabitants.

Previously declared epidemics had already proved their potential to generate psychological impact (Chan‐Yeung, 2004), but unfortunately none has had the magnitude of the current pandemic. The global situation of accumulating cases and deaths and the extensive media coverage added to the individual fear of contagion, alongside with the fear of forced social transformation in the future, are all elements that might contribute to the detriment in mental health (Aiello et al., 2020; Stella et al., 2020).

Although COVID‐19 has affected the same number of men and women, gender differences have been identified in terms of fatality rates ((CDC) C for DC and P, 2020) suffering men a worse prognosis and a higher risk of death (Spagnolo et al., 2020). On the other hand, COVID‐19 has also become a potent stressor with people experiencing fear and isolation over a long period of time, leading to an increased vulnerability to anxiety, depression, and acute stress that affect women more than men (Mauvais‐Jarvis et al., 2020). Proof of this is the higher prevalence and severity of symptoms of anxiety, depression, and acute stress observed in females during the initial phase of the pandemic (Liu et al.,).

According to the international psychiatric classifications, a traumatic event is defined as the exposure to death, threatened death, actual or threatened serious injury, or actual or threatened sexual violence (APA, 2013). Hence, this pandemic could be considered a trauma. Research has pointed out that women are more likely than men to develop distress after being exposed to trauma (Tolin & Foa, 2006). However, this gender difference is not absolute, when examining specific very high‐risk events for the development of post‐traumatic stress disorder, the relative risk equalizes or even reverses (Kessler et al., 1995).

To the best of our knowledge, there are few published studies addressing the gendered impact of the COVID‐19 outbreak in a country like Spain, where mortality remains as one of the highest worldwide. With this purpose, we aimed to explore the differential presence of symptoms of anxiety, depression, and acute stress between men and women, and to study the relationship between these symptoms and two environmental variables, coexistence and violence.

## 2 METHODS

A total of 520 men and 1,115 women, aged from 18 to 84 (mean 40.4 ± 14.1 years), participated in this cross‐sectional study previously published by our group (García‐Fernández et al., 2020), based on a national online survey applying an **e**xponential nondiscriminative snowball sampling**.** The questionnaire was published on the hospital website and distributed by social networks to different geographical regions with the aim of reaching a representative sample of the Spanish population.

Up to 2,710 participants completed the self‐reported questionnaire from March 29 to April 5, 2020, which covers the peak of the SARS‐CoV‐2 infection in Spain. Anyone with access to social networks who consented to participate and was over 18 completed the questionnaire. Of total respondents, healthcare workers and people with a current or past mental disorder were excluded for the present study as they have been considered especially vulnerable population groups for emotional reactions to COVID already predisposed to experience more stress and have been analyzed in a different study (PsyCOVID San Juan imas12).

Informed consent was provided, and confidentiality was assured. The study was approved by Hospital de San Juan's local ethics committee.

Sociodemographic information on age, gender, and occupation was required, as well as whether responders had lived alone or with other people and had experienced situations of violence, either emotional or physical abuse, during the pandemic period.

To assess symptoms of anxiety and depression, we included the Hamilton Anxiety Scale (HARS) (Hamilton, 1959; Lobo et al., 2002) and the Beck Depression Inventory (BDI) (Bech, 1988; Vazquez & Sanz, 1999), respectively. For reporting the presence of acute stress, we adapted ad hoc for this study the clinical criteria for the diagnosis of Acute Stress Disorder (Acute stress disorder inventory—ASDI) of the Diagnostic and Statistical Manual of Mental Disorders (DSM‐5) (APA, 2013). We developed a list of symptoms to be applied as self‐reported questionnaire with a dichotomous answer (yes/no). In addition, the score of total affirmative responses to each of the items was recorded.

Differences between males and females in clinical variables were tested using one‐way analysis of variance corrected for age and chi‐squared tests as appropriate. Then, the ANOVAs were repeated including the following sociodemographic variables: coexistence (living alone versus with other people), having experienced violence (yes versus no), economic losses (yes versus no), unemployment (yes versus no), COVID‐19 infection (confirmed/suspected/discarded), and having experienced the passing of a loved one (yes versus no). All these variables were asked referring to the pandemic period. The analyses presented have been corrected for age. Bonferroni corrections for multiple comparisons have been applied when appropriate. All statistical analyses were considered statistically significant when *p* < .05.

## 3 RESULTS

### 3.1 Relationship between gender and clinical variables

Regarding anxiety symptoms (*F* = 56.2, *p* ******< .001), males had significant lower HARS scores (*M* = 14.1, *SD* = 9.8) than females (*M* = 18.4, *SD* = 10.2). In addition, results showed that males had significant lower BDI scores than females (*F* = 16.6, *p *< .00; *M* = 3.4, *SD* = 3.9 and *M* = 4.5, *SD* = 4.3, respectively). In the same line, when a cutoff syndrome score of 4 (absent or minimal versus. mild/moderate/severe depression) is applied, a weaker depressive syndrome in males was observed (χ^2^ = 25.5, *p *< .00; 28.1% males versus. 39.9% females). Finally, in ASDI scores (*F* = 39.0, *p *< .001), males had significantly lower ASDI scores than females (*M* = 3.6, *SD* = 2.9 and *M* = 4.7, *SD* = 3.1, respectively).

### 3.2 Relationship between gender and environmental variables

A significant interaction Gender × Coexistence was found in anxiety (*F* = 56.2, *p *< .001). Specifically, while there were no differences in males living alone or living with other people, females living alone showed significantly higher scores than those who lived with other people. No other variables had a differential impact on anxiety depending on the gender of the respondent: violence (*F* = 0.27, *p *= .60), economic losses (*F* = 0.002, *p*=.97), unemployment (*F* = 0.42, *p *= .52), COVID‐19 infection (*F* = 0.54, *p *= .59), or death of a loved one (*F* = 0.20, *p *= .66).

According to depressive symptoms, the interaction Gender x Violence was marginally significant (*F* = 3.3, *p *= .07). There were no differences in BDI scores between males who had experienced violence and those who had not. However, females who had experienced domestic violence had significantly higher levels of depressive symptoms than those who had not. No other variables had a differential impact on depressive symptoms depending on the gender of the respondent: coexistence (*F* = 0.20, *p *= .66), economic losses (*F* = 0.10, *p *= .75), unemployment (*F* = 0.13, *p *= .71), COVID‐19 infection (*F* = 1.27, *p *= .28), or death of a loved one (*F* = 0.61, *p *= .44).

In the case of ASDI scores, the interaction Gender x Coexistence was marginally significant (*F* = 3.52, *p *= .06). The mean difference in ASDI scores between males and females who live alone (women measuring higher) is more than twice compared to the difference between those who do not live alone. No other variables showed an interaction effect with gender: violence (*F* = 0.96, *p*=.33), economic losses (*F* = 1.21, *p*=.27), unemployment (*F* = 0.16, *p*=.69), COVID‐19 infection (*F* = 1.70, *p*=.18), or death of a loved one (*F* = 0.22, *p*=.64). (Mean and *SD* of all these analyses are presented in Table 1).

**Table 1:** Mean and standard deviations of the psychological variables (HARS, BDI, and ASDI) as function of gender and the sociodemographic variables included in the analyses

		Males			Females		
		HARS	BDI	ASDI	HARS	BDI	ASDI
Coexistence	No	12.9 (10.1)	3.5(3.8)	3.1(2.8)	20.2(11.8)	4.7(4.4)	5.0(3.4)
	Yes	14.3 (9.8)	3.4 (3.9)	3.7 (2.9)	18.2 (10.0)	4.5 (4.3)	4.7 (3.1)
Violence	No	13.9 (9.7)	3.4 (3.9)	3.5 (2.8)	18.2 (10.0)	4.4 (4.2)	4.7 (3.1)
	Yes	20.0 (10.7)	4.5 (2.8)	6.5 (3.6)	25.9 (12.7)	7.9 (6.5)	6.9 (3.2)
Economic losses	No	2.8 (3.4)	2.8 (3.4)	2.9 (2.5)	4.0 (3.9)	4.0 (3.9)	4.3 (3.0)
	Yes	4.1 (4.4)	4.1 (4.4)	4.4 (3.1)	5.3 (4.7)	5.3 (4.7)	5.4 (3.2)
Unemployment	No	14.4 (9.7)	4.3 (4.2)	3.8 (2.9)	18.3 (10.2)	5.2 (4.6)	4.8 (3.2)
	Yes	14.0 (9.8)	3.1 (3.8)	3.5 (2.9)	18.4 (10.2)	4.0 (4.0)	4.6 (3.1)
COVID−19	No	13.5 (9.4)	3.3 (3.9)	3.5 (2.8)	18.0 (10.0)	4.5 (4.3)	4.7 (3.1)
	Suspected	18.5 (11.7)	4.1 (4.3)	4.3 (3.2)	21.5 (10.4)	4.8 (3.9)	5.3 (3.1)
	Yes	21.0 (0.0)	1.0 (0.0)	1.0 (0.0)	30.3 (15.0)	7.3 (4.4)	7.4 (4.5)
Death of a loved one	No	14.0 (9.8)	3.4 (3.9)	3.6 (2.8)	18.3 (10.2)	4.6 (4.3)	4.7 (3.1)
	Yes	15.9 (9.3)	3.5 (4.2)	4.1 (3.2)	19.4 (9.8)	4.2 (3.5)	4.8 (3.1)

## 4 DISCUSSION

The first aim of the present study was to explore the differential presence of symptoms between men and women during the peak of the COVID‐19 outbreak in Spain. Findings indicate, as has also been observed in Europe and China (Gebhard et al., 2020; Liu et al.,; Wang et al., 2020), that women present greater severity in symptoms of anxiety, depression, and acute stress, which denotes an increase in arousal response to stress in females (Bangasser & Wicks, 2017) supporting sex differences in stress response systems, also during COVID‐19. Hence, these results seem to point out that although the objective risk, due to morbidity and mortality ((CDC) C for DC and P, 2020) of the COVID‐19 pandemic, is significantly greater for men, the emotional response is higher in women, which evidences the existence of other factors, beyond verified data on severity, influencing the emotional response.

The study further demonstrates that women living alone show more severe levels of anxiety, a fact that has not been observed in men, supporting the existence of gender differences that remain in the response to COVID and that may have to do with the predominant role of women as family caregivers and the greater susceptibility to social isolation (Gebhard et al., 2020; Spagnolo et al., 2020). In the same line, having experienced situations of domestic violence during the period of the pandemic is associated, only in women, with greater depressive symptoms. These findings add valuable information and support the gender differences found in relation to the concerns during COVID‐19 of men and women, focused on the effects on the economy and society in men and on the family health and well‐being in women (Van der et al., 2020).

The main strength of the study includes data collection from a wide general population sample during a particularly difficult period of infection in Spain, but some limitations must be pointed out. First, response bias exist as a voluntary online self‐administered survey was applied, second, there is a difference in the number of men and women who have responded to the survey, and to end, an anxiety scale initially designed for the evaluation of people with mental disorders has been applied as well as a scale to measure acute stress based on the DSM 5 criteria still in the validation process.

To the best of our knowledge, this is the first time that symptoms of anxiety, depression, and acute stress after the outbreak of COVID‐19 are assessed in Spain in a great sample, incorporating both a broad representation of the general population and a gender perspective. The response to coronavirus appears to be different between men and women. Moreover, loneliness and violence specifically worsen the emotional state in women. These results emphasize the need to address the gender impact of outbreaks, as well as the benefit of paying special attention to women living alone and those who suffer domestic violence to ensure their emotional well‐being.

## CONFLICT OF INTEREST

Dr. R. Rodriguez‐Jimenez has been a consultant for, spoken in activities of, or received grants from Instituto de Salud Carlos III, Fondo de Investigación Sanitaria (FIS), Centro de Investigación Biomédica en Red de Salud Mental (CIBERSAM), Madrid Regional Government (S2010/ BMD‐2422 AGES; S2017/BMD‐3740), JanssenCilag, Lundbeck, Otsuka, Pfizer, Ferrer, Juste, Takeda, Exeltis, Angelini, and Casen‐Recordati. All other authors declare that they have no conflict of interest.

## AUTHOR CONTRIBUTIONS

LGF and RRJ designed the study and wrote the protocol; LGF, PDLR, and MMG carried the study out; VRF and SPU undertook the statistical analysis; all authors contributed and have approved the final manuscript.

### Peer Review

The peer review history for this article is available at https://publons.com/publon/10.1002/brb3.1934.

## Data Availability

The data that support the findings of this study are available on request from the corresponding author. The data are not publicly available due to privacy or ethical restrictions.
